# A Case of a Large Brain Metastasis: Time for Multidisciplinary Consultation

**DOI:** 10.7759/cureus.74468

**Published:** 2024-11-26

**Authors:** Alis Guberinic, Berber Piet, Frederick Meijer, Anja Gijtenbeek, Mark ter Laan

**Affiliations:** 1 Department of Neurosurgery, Radboud University Medical Center, Nijmegen, NLD; 2 Department of Pulmonology, Radboud University Medical Center, Nijmegen, NLD; 3 Department of Medical Imaging, Radboud University Medical Center, Nijmegen, NLD; 4 Department of Neurology, Radboud University Medical Center, Nijmegen, NLD

**Keywords:** brain metastases, multidisciplinary consultation, non-small cell lung carcinoma, pseudoprogression, tumor markers

## Abstract

Patients with complex diseases are mostly treated in a multidisciplinary setting. The impact of multidisciplinary care cannot be emphasized enough as it has the potential to significantly increase survival and, in some cases, help avoid a risky treatment approach. The aim of this case illustration is to emphasize the importance of multidisciplinary treatment and learn from the different approaches that can be made while treating such patients. This case describes the clinical course of a 40-year-old female patient with metastatic non-small cell lung carcinoma (NSCLC), TNM classification cT3N3M1c. Although she had clinical and radiological signs of brain herniation, a risky neurosurgical intervention was prevented due to an extensive multidisciplinary approach. Moreover, the important role of multidisciplinary consultation, imaging, and tumor markers in identifying pseudoprogression in the increasingly complex treatment of metastatic NSCLC is also demonstrated. In conclusion, a multidisciplinary approach is a crucial step in the growing complex treatment strategies for patients with brain metastases of NSCLC.

## Introduction

Management of primary and metastatic tumors of the central nervous system (CNS) involves coordination of medical and surgical treatment of these conditions as well as associated complications, including complications of the therapies themselves. Frequent communication and collaboration across medical specialties are required.

Of patients with a malignant solid tumor, 20% develop brain metastases [1]. Lung carcinomas most often metastasize to the brain and approximately 50% of brain tumors are due to lung cancer [2,3]. Early diagnosis and opting for the best possible treatment can be challenging in patients with brain tumors. Various considerations are important, namely the condition of the patient, neurological symptoms, the systemic treatment options based on immunohistochemical and molecular tumor characteristics, systemic disease activity, number of metastases, volume and location of the brain metastases, and the wishes and expectations of the patient. Therefore, experts in various fields are required if all considerations are to be accounted for when strategizing for the best treatment option. By considering the different opinions in a multidisciplinary consultation, unnecessary diagnostics can be prevented and survival can be improved. It can also decrease the time of diagnosis and treatment and in some cases even prevent a risky treatment [4,5].

Furthermore, in clinical practice, it can be debated whether symptomatic brain metastases should be treated locally (e.g. surgery or stereotactic radiotherapy), or whether a possible effect of the systemic therapy can be awaited. It is therefore essential that treatment decisions regarding brain metastases in lung cancer are contemplated in a neuro-oncological multidisciplinary consultation.

This report describes a case that shows the importance of multidisciplinary consultation. It also demonstrates the importance of diagnosis pseudoprogression for patients who are treated with immunotherapy. In such cases, treatment with corticosteroids is usually sufficient to reduce the mass effect of the perilesional edema. Resection then becomes an option for when the mass effect cannot be controlled with corticosteroids [6].

## Case presentation

A 40-year-old female patient presented with a dry cough for the past six weeks and progressive pain between the shoulder blades in May 2022. Despite her relatively young age and the lack of risk factors such as smoking, additional research showed a stage IV human epidermal growth factor receptor 2 (HER2) exon 20 insertion non-small cell lung cancer (NSCLC), with extensive metastases in the lymph nodes, liver, skeleton, and cerebrum (TNM classification cT3N3M1c). A biopsy was taken from two supraclavicular lymph nodes. Pathological analysis demonstrated adenocarcinoma, with a PD-L1 expression of one percent and a HER2 exon 20 insertion mutation. Computed tomography (CT) and magnetic resonance imaging (MRI) with contrast of the brain showed at least 18 lesions suggestive of brain metastases. The largest lesion was in the left temporal lobe which was 46 mm in diameter. Different oncomarkers were investigated at the time of diagnosis, namely cyfra-21 which was 48.6, cancer antigen (CA)-125 was 130 and carcinoembryonic antigen (CEA) was 240. She was treated with first-line palliative chemo-immunotherapy according to the Dutch guidelines, which consisted of carboplatin, pemetrexed, and pembrolizumab.

**Figure 1:**
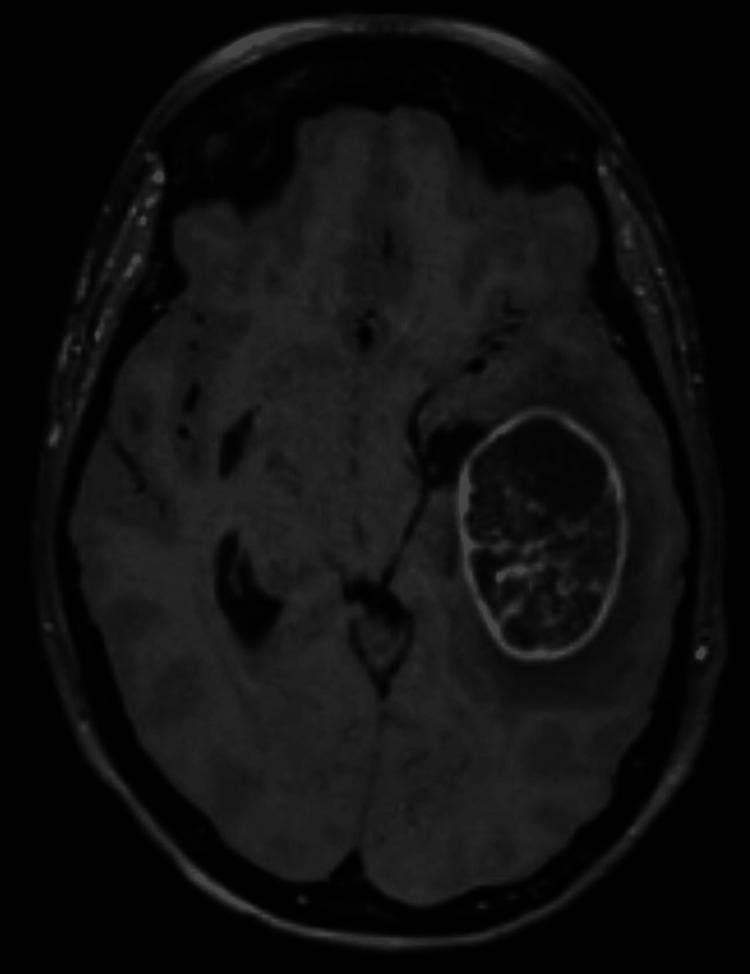
MRI cerebrum T1 sequence with contrast six days after starting chemoimmunotherapy showing left temporal lobe brain metastasis with midline shift and uncal herniation. The hypodensity on the ventral part of the lesion is suggestive for central necrosis since it does not enhance. This is suggestive for tumor response.

Six days after her first treatment she was admitted to the emergency department with progressive headache, nausea, vomiting, and dizziness. In the emergency department, the patient had an expressive aphasia, and was awake but bradyphrenic with no further focal neurological deficit. We started with dexamethasone 8 mg once a day due to these symptoms. The laboratory examination showed no abnormalities except for slightly elevated liver enzymes. Due to the suspicion of progression of the brain metastases, a head CT and subsequently an MRI were performed. Both showed a significant increase in perifocal edema in the left temporal lobe with mass effect and therefore subfalcine and uncal herniation (Figure 1).

There was a clear surgical indication due to the clinical and radiological signs of brain herniation. Nevertheless, a multidisciplinary meeting was scheduled, and it was concluded that the symptoms were either due to the tumor or pseudoprogression (in this case extensive necrosis leading to edema). To distinguish between these, the tumor markers in the peripheral blood were examined. A decrease of 44.7 was seen in Cyfra 21-1, making response to therapy with pseudoprogression more likely. This in combination with the relative fast-onset of neurological symptoms with the start of chemo-immunotherapy and the extensive necrosis and edema on the MRI was sufficient reason to await the effect of dexamethasone. The patient improved clinically during admission and a new multidisciplinary consultation was scheduled. The consensus was that further effects of the systemic therapy could be awaited.

**Figure 2:**
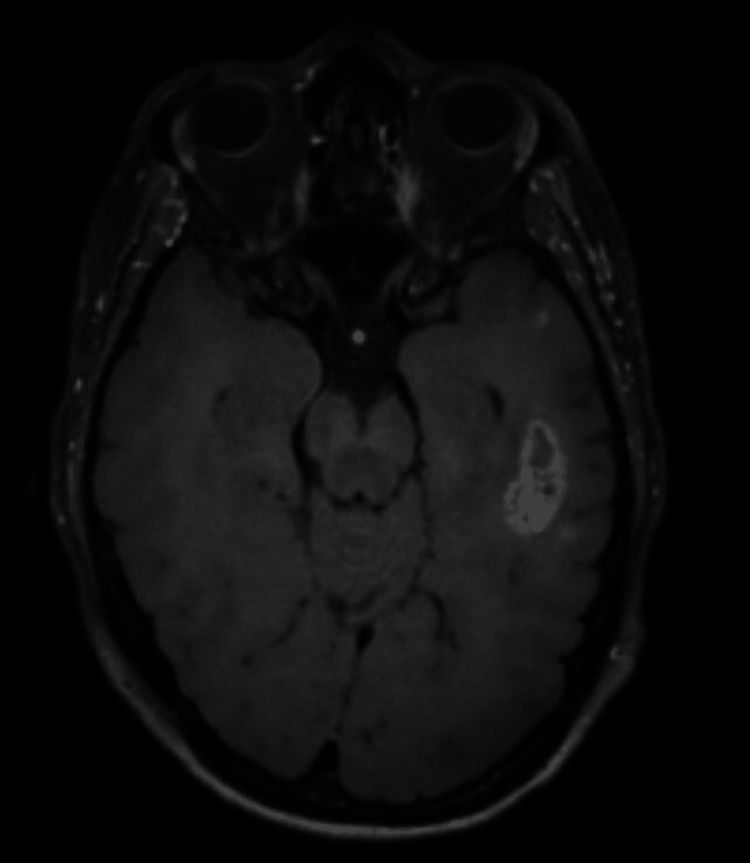
MRI cerebrum T1 sequence with contrast showing left temporal lobe brain metastasis, five weeks after start of chemoimmunotherapy with evident regression of left temporal brain metastasis.

Shortly thereafter, the patient was discharged from the hospital with dexamethasone 4 mg once a day maintenance. The follow-up MRI showed significant tumor regression five weeks after the start of chemo-immunotherapy (Figure 2). The systemic response showed a significant decrease in the size of the two lymph nodes, metastasis in the liver, and the primary tumor in the right lung. However, after three months, the follow-up MRI showed an increase in the size of the brain lesions. The following months showed even more progression of the disease although the chemo-immunotherapy was continued.

## Discussion

This case illustrates the important role of multidisciplinary consultation and to identify pseudoprogression in the increasingly complex treatment of metastatic NSCLC. The impact of multidisciplinary care cannot be emphasized enough as it has the potential to significantly increase survival [3]. Early discussion in the treatment of an individual patient can prevent unnecessary diagnostic investigations and interventions and save valuable time [4]. This specifically is of great importance when treating patients with brain metastasis since these patients can deteriorate quickly. A group of experts in their field can improve the quality of cancer care by preventing and diminishing treatment side-effects and when present can be acted on quickly [4,7].

There would have been an indication for surgery in this case since the patient showed clinical and radiological signs of herniation. However, by involving various professionals, pseudoprogression was diagnosed early. This ultimately resulted in the avoidance of major neurosurgery in an eloquent area with a significant risk of aphasia and therefore potentially major impact on quality of life. We are aware that most oncological units have their scheduled multidisciplinary meetings. However, we advocate for non-scheduled meetings to discuss a complex case when presenting with acute neurological symptoms as this reduces the time to diagnosis and/or start of treatment [6]. This is specifically important with pseudoprogression since it can be challenging to differentiate from true tumor progression noted clinically or radiographically, thereby complicating management decisions [4]. Pseudoprogression is a phenomenon where the tumor increases in size due to infiltration of lymphocytes and consequently perilesional edema [8]. This can cause major health problems in patients with brain metastasis and therefore should be managed as quickly as possible. However, diagnosing pseudoprogression poses a lot of challenges as it is difficult to differentiate from progression with the current imaging techniques [8,9].

We used tumor markers like Cyfra-21 to aid the imaging diagnostics. Cyfra 21-1 is a cytokeratin-19 that is soluble in serum and is specific for NSCLC [10]. It therefore could be used to understand the likeliness of tumor progression, depending on whether it raises or drops from baseline. The imaging techniques and laboratory findings should always be taken together with the clinical course in diagnosing pseudoprogression. It is therefore essential to have experts in their field in a multidisciplinary meeting to interpret these results. This results in a well-defined therapy, which can be started efficiently and quickly.

It is to be noted that during the follow-up period after three months we saw progression of the tumor even with chemo-immunotherapy. The disease progressed even further in the following months.

## Conclusions

A multidisciplinary discussion is crucial for optimal diagnosis and treatment decision-making in complex oncological cases, also in case of a neurological emergency. It is important to involve all relevant experts in respective fields in the increasingly complex oncological treatment landscape. Early discussion in the treatment of an individual patient can prevent unnecessary diagnostic investigations and interventions and save valuable time. As a result, everyone can contribute ideas from their own expertise and thus realize the best treatment strategy for the patient. Also, the use of tumor markers can help in the diagnosis of pseudoprogression, which can help in decision-making for the best treatment option.
